# Using patient-held records to evaluate contraceptive use in Malawi

**DOI:** 10.2471/BLT.14.145623

**Published:** 2015-08-31

**Authors:** Aisha NZ Dasgupta, Ruth Ngwalo, Keith Branson, Levie Gondwe, Frank Taulo, Bagrey Ngwira, Basia Zaba, Amelia C Crampin

**Affiliations:** a London School of Hygiene & Tropical Medicine, Keppel Street, London, WC1E 7HT, England.; b District Health Office, Karonga, Malawi.; c Karonga Prevention Study, Chilumba, Malawi.; d College of Medicine, University of Malawi, Blantyre, Malawi.

## Abstract

**Objective:**

To investigate a method of using patient-held records to collect contraception data in Malawi, that could be used to explore contraceptive discontinuation and method switching.

**Methods:**

In 2012, all 7393 women aged 15 to 49 years living in the area covered by the Karonga demographic surveillance site were offered a family planning card, which was attached to the woman’s health passport – a patient-held medical record. Health-care providers were trained to use the cards to record details of contraception given to women. During the study, providers underwent refresher training sessions and received motivational text messages to improve data completeness. After one year, the family planning cards were collected for analysis.

**Findings:**

Of the 7393 eligible women, 6861 (92.8%) received a family planning card and 4678 (63.3%) returned it after one year. Details of 87.3% (2725/3122) of contacts between health-care providers and the women had been recorded by health-care providers on either family planning cards or health passports. Lower-level health-care providers were more diligent at recording data on the family planning cards than higher-level providers.

**Conclusion:**

The use of family planning cards was an effective way of recording details of contraception provided by family planning providers. The involvement of health-care providers was key to the success of this approach. Data collected in this way should prove helpful in producing accurate estimates of method switching and the continuity of contraceptive use by women.

## Résumé

**Objectif:**

Examiner une méthode consistant à utiliser les dossiers tenus par les patients pour recueillir des données sur la contraception au Malawi qui pourraient permettre d'analyser les interruptions et les changements de méthodes de contraception.

**Méthodes:**

En 2012, l'ensemble des 7393 femmes âgées de 15 à 49 ans de la région couverte par le système de surveillance démographique de Karonga s'est vu proposer une carte de planification familiale, attachée à leur carnet de santé – un dossier médical tenu par les patients. Les professionnels de santé ont été formés à utiliser ces cartes pour noter les renseignements sur la contraception prescrite aux femmes. Au cours de l'étude, ils ont assisté à des séances de remise à niveau et ont reçu des SMS d'incitation en vue d’améliorer l'exhaustivité des renseignements inscrits. Au bout d'un an, les cartes de planification familiale ont été recueillies pour analyse.

**Résultats:**

Sur les 7393 femmes éligibles, 6861 (92,8%) ont reçu une carte de planification familiale et 4678 (63,3%) l'ont restituée au bout d'un an. Des renseignements sur 87,3% (2725/3122) des contacts entre les professionnels de santé et les femmes avaient été consignés par les professionnels de santé sur les cartes de planification familiale ou les carnets de santé. Les professionnels de santé de niveau inférieur étaient plus appliqués à inscrire les renseignements sur les cartes de planification familiale que ceux de niveau plus élevé.

**Conclusion:**

L'utilisation de cartes de planification familiale s'est révélée un moyen efficace de consigner les renseignements sur la contraception prescrite par les prestataires de services de planification familiale. L'implication des professionnels de santé était indispensable à la réussite de cette démarche. Les données recueillies de cette manière devraient permettre de faire des estimations précises des changements de méthodes et de la continuité du recours aux contraceptifs par les femmes.

## Resumen

**Objetivo:**

Investigar un método para hacer uso de las historias clínicas de los pacientes para recopilar datos sobre los anticonceptivos en Malawi, los cuales podrían utilizarse para estudiar la eliminación de los anticonceptivos y los cambios de métodos.

**Métodos:**

En 2012, se ofreció a todas las 7.393 mujeres de entre 15 y 49 años que vivían en la zona cubierta por los puestos de vigilancia demográfica de Karonga una cartilla de planificación familiar, que se adjuntaba a su pasaporte sanitario (la historia clínica del paciente). Se enseñó a los profesionales sanitarios a hacer uso de las historias para documentar detalles sobre los anticonceptivos administrados a las mujeres. A lo largo del estudio, los profesionales se sometieron a sesiones formativas de actualización y recibieron mensajes de texto motivacionales para mejorar el cumplimiento total de los datos. Después de un año, se recogieron las cartillas de planificación familiar para analizarlas.

**Resultados:**

De las 7.393 mujeres que cumplían los requisitos, 6.861 (92,8%) recibieron una cartilla de planificación familiar y 4.678 (63,3%) la devolvieron al cabo de un año. Los detalles del 87,3% (2.725/3.122) de las visitas entre los profesionales sanitarios y las mujeres fueron documentados por los primeros en las cartillas de planificación familiar o bien en los pasaportes sanitarios. Los proveedores de atención sanitaria de bajo rango fueron más minuciosos a la hora de documentar los datos en las cartillas de planificación familiar que los de alto rango.

**Conclusión:**

El uso de las cartillas de planificación familiar fue una forma efectiva de registrar los detalles de los anticonceptivos administrados por los proveedores de planificación familiar. La implicación de los proveedores de atención sanitaria fue clave para el éxito de este enfoque. La información recopilada de esta forma deberá ser de ayuda a la hora de producir estimaciones rigurosas sobre si existen cambios de métodos y continuidad en el uso de anticonceptivos por parte de las mujeres.

## ملخص

**الغرض:**

إجراء استقصاء لتحديد طريقة لاستخدام السجلات التي يملكها المرضى لجمع البيانات المتعلقة بمنع الحمل في ملاوي، حيث يمكن الاستعانة بهذه الطريقة في استكشاف معلومات عن التوقف عن استعمال موانع الحمل والتحول لاستخدام طرق بديلة.

**الطريقة:**

تم تقديم بطاقة تنظيم الأسرة إلى جميع النساء اللاتي تتراوح أعمارهن بين 15 و49 عامًا والبالغ عددهن 7393 ممن يقيمون بالمنطقة التي تشملها خدمة موقع الترصد الديموغرافي في كارونجا، وذلك في عام 2012، حيث تُرفق بالبطاقة الصحية الخاصة بالمرأة – والتي تمثل سجلًا طبيًا يملكه المريض. وقد تلقى مقدمي خدمات الرعاية الصحية تدريبًا على استخدام هذه البطاقات التي تم إعطاؤها للنساء لتسجيل تفاصيل منع الحمل. وأثناء الدراسة حضر مقدمو هذه الخدمات جلسات تدريبية لتجديد المعلومات، كما تلقوا رسائل نصية تحفيزية لرفع مستوى اكتمال البيانات. وبعد مرور عام واحد، تم جمع بطاقات تنظيم الأسرة لتحليلها.

**النتائج:**

حصلت 6861 (92.8%) امرأة من بين 7393 امرأة تنطبق عليها الشروط، على بطاقة تنظيم الأسرة، بينما أرجعت 4678 (63.3%) منهن البطاقة بعد مرور عام واحد. وسجّل مقدمو خدمات الرعاية الصحية تفاصيل 87.3% (2725/3122) من مرات التواصل بينهم وبين النساء فيما يتعلق إما ببطاقات تنظيم الأسرة أو البطاقات الصحية. وتحلى مقدمو خدمات الرعاية الصحية في المستويات الأقل بهذا المجال بمزيد من الجدية في تسجيل البيانات المتعلقة ببطاقات تنظيم الأسرة مقارنةً بمقدمي خدمات الرعاية الصحية في المستويات الأعلى بهذا المجال.

ا **لاستنتاج ** كان استخدام بطاقات تنظيم الأسرة يمثل إحدى الطرق الفعالة لتسجيل التفاصيل المتعلقة بمنع الحمل التي يتم الحصول عليها عن طريق مقدمي خدمات الرعاية الصحية القائمين على تنظيم الأسرة. وكان إشراك مقدمي خدمات الرعاية الصحية في ذلك عنصرًا رئيسيًا لنجاح هذا النهج. ويُفترض أن تساعد البيانات التي تم جمعها بهذه الطريقة في الخروج بتقديرات دقيقة لدرجة التحول لاستخدام طرق بديلة واستمرار النساء في استخدام موانع الحمل. ****

## 摘要

**目的:**

旨在调查利用患者保存的记录来收集马拉维境内避孕数据的方法，该方法可以用来探索避孕药具的停用和方法转换。

**方法:**

2012 年，居住在卡龙加县人口监测站点覆盖范围内的所有 15 至 49 岁女性（共 7393 名）都收到了计划生育卡，该卡附在女性的医疗记录册——患者保存的医疗记录内。 医疗服务提供者接受了培训，使用该卡记录女性使用避孕药具的详细情况。在研究过程中，提供者接受了进修培训课，并且收到了激励性的短信，以提高数据完整性。 一年之后，收集计划生育卡进行分析。

**结果:**

在 7393 名符合条件的女性中，有 6861 (92.8%) 名收到了计划生育卡，4678 (63.3%) 名在一年后返还了计划生育卡。 医疗服务提供者在计划生育卡或医疗手册上记录了医疗服务提供者与 87.3% (2725/3122) 女性之间的联系详情。 低级别的医疗服务提供者比高级别的更加勤于在计划生育卡上记录数据。

**结论:**

计划生育卡的使用是记录计划生育提供者所提供避孕详情的一种有效方法。 医疗服务提供者的参与是该方法成功的关键。 用这种方法收集的数据对准确评估方法转换和女性使用避孕药的持续性颇有助益

## Резюме

**Цель:**

Изучить метод ведения пациентами записей с целью сбора информации о методах контрацепции, принятых в Малави. Получить информацию для исследования прекращения применения контрацептивных средств и перехода от одного метода контрацепции к другому.

**Методы:**

В 2012 году жительницам региона, который находится в ведении участка демографических наблюдений Каронга, предложили так называемые карты планирования семьи. В исследовании приняли участие 7393 женщины в возрасте от 15 до 49 лет. Карты планирования семьи прилагались к «паспорту здоровья женщины» — медицинской карте, которую ведет сама пациентка. Медицинских работников обучили использованию этих карт для фиксации подробной информации о том, какие контрацептивные средства они предлагали женщинам. Во время исследования медицинские работники проходили повторное обучение и получали мотивационные текстовые сообщения с целью добиться большей полноты данных. Спустя год карты планирования семьи были собраны для анализа.

**Результаты:**

Из 7393 женщин, которые смогли принять участие в исследовании, 6861 получила карту планирования семьи (92,8%) и 4678 (63,3%) женщин вернули эти карты врачам спустя год. 87,3% (2725 из 3122 человек) пациентов располагали подробными сведениями о посещении врачей. Данные сведения были внесены самими врачами в карту планирования семьи или паспорт здоровья женщины. Следует отметить, что медицинские работники низшего звена были более прилежны в том, что касается внесения данных в карты планирования семьи, нежели медработники более высокого уровня.

**Вывод:**

Использование карт планирования семьи оказалось эффективным методом фиксации подробных сведений о том, какого рода противозачаточные средства предлагались женщине ее врачами. В этом исследовании участие и заинтересованность самих медицинских работников играли ключевую роль. Собранные таким образом данные полезны для точных оценок необходимости продолжения использования женщиной противозачаточных средств или перехода с одного метода контрацепции на другой.

## Introduction

Access to contraceptive services is important, not only because of the direct effect on reproductive health, but also because contraceptive use may lead to indirect improvements in general health and socioeconomic outcomes.^1^^–^^3^ Contraceptive use is one of the key proximate determinants of reduced fertility,^4^ which is, in turn, associated with economic development indicators. In Malawi, there has been a remarkable increase in contraceptive use over the past two decades: 7% of married women reported using a modern contraceptive method in 1992 compared with 42% in 2010.^5^ Paradoxically, fertility remains high. In 2010, women in Malawi bore on average 5.7 children and many pregnancies were unintended or occurred sooner than desired.^5^ A reason for this paradox could be overreporting in cross-sectional surveys: a woman might report using contraception, even if in reality she has missed or delayed family planning appointments and has discontinued a short-term method. Contraceptive discontinuation and switching of methods are key factors because, as desired family size decreases and contraceptive use increases, the effectiveness and duration of contraception become increasingly important determinants of fertility, unintended pregnancies and induced abortions.^6^

Data on contraceptive use come from a variety of sources, including routine health facility records, cross-sectional surveys and retrospective surveys. Demographic and health surveys now include a contraceptive calendar, which captures self-reported data on contraceptive use, pregnancy, childbirth, breastfeeding and pregnancy termination for each calendar month in the five years before the survey interview. Although calendar data do not suffer from the problem of loss-to-follow-up, there is a selection bias as only women who survive until the interview can report, and there are likely to be recall issues. One study from Bangladesh found poor consistency between reports given in a baseline interview and reports given in a follow-up survey in which women were asked to describe retrospectively their contraceptive use during the month covered by the baseline survey;^7^ consistency was especially poor for women with complex reproductive histories. Single-country studies on contraceptive discontinuation and switching, based on data from sources other than demographic and health surveys, are uncommon because such data tend to be difficult to collect and analyse. Thus, most research has been based on cross-country comparative reports, which often used data from the calendar section of the demographic and health survey questionnaire.^6^^,^^8^^–^^15^ Using 2004 data from the Malawi Demographic and Health Survey, researchers found high contraceptive discontinuation rates in the country.^12^ Compared to 17 other developing countries, Malawi had the lowest proportion of women who switched to another modern contraception method within three months of method-related discontinuation, which suggests that switching behaviour (using another method) following discontinuation is poor.^12^ With the exception of the retrospective calendar, however, conventional assessments of contraceptive use are not able to capture switching or discontinuation. There is, therefore, a need for a prospective method for collecting more reliable data on contraceptive episodes.

In Malawi, a range of contraceptive methods are provided through different mechanisms (i.e. public or private through clinics or outreach)^16^ by various health-care providers: clinical officers, nurses, medical assistants, health surveillance assistants and volunteer community-based distribution agents.^17^^–^^19^ Women are expected to carry a health passport (a patient-held medical record) with them when they use health-care services. Some health passports – but not all – contain a dedicated family planning page on which health-care providers can record details of the contraceptive services provided.

The Karonga Prevention Study in northern rural Malawi operates a demographic surveillance site that covered 36 524 individuals at the end of 2012.^20^ Recent studies nested within the site have focused on adult human immunodeficiency virus (HIV) infection, sexual and reproductive behaviour and childbearing intentions.^21^^–^^24^ The contraceptive services in the study area are managed by the district family planning coordinator at the district health office. The demographic surveillance site provides an ideal setting for observational studies of contraception because the site has close links with health facilities and other providers of contraception and there is an opportunity to collaborate with the district family planning team. Also, new data on contraception use can be linked to the Karonga prevention study database, which holds current demographic and socioeconomic data on residents in the site. The database also provides information about, for example, childbearing intentions, marital history, parity and HIV status. Linking the data makes it possible to explore explanatory variables.

It has been suggested that patient-held medical records could play an important role in monitoring the continuity of health service use.^25^^,^^26^ Here we investigated a method for using patient-held records – a so-called family planning card – to collect contraceptive data. This approach captures data recorded by providers and could form the basis of a prospective longitudinal data set. Such a data set would make it possible to study the continuity of contraceptive use and switching of methods and could serve to validate cross-sectional estimates of contraceptive use.

## Methods

All women aged 15 to 49 years living in the area covered by the Karonga demographic surveillance site between January and April 2012 were eligible to participate in the family planning study. Consenting women were offered a family planning card. Subsequently, when a woman accessed family planning services, the health-care provider recorded details of her visit on the card. After one year, the family planning cards were collected by Karonga prevention study staff and data were linked to the database using unique, identifying information for each woman – and we analysed the data. Ethical approval was obtained from research and ethics committees at the London School of Hygiene & Tropical Medicine, United Kingdom of Great Britain and Northern Ireland and the College of Medicine, Malawi.

Before starting the study, approval was sought from the Wasambo Traditional Authority Chief, local village headmen and headwomen, the Karonga Area Development Committee and the Karonga District Health Management Team. Subsequently, the aims and data collection methods of this and two other studies were explained through 16 community sensitization activities involving local dance, song troupes and the study staff. These activities were undertaken to address potential misconceptions among the local community, to increase understanding of the nature of the study and, thereby, to improve the quality of the consent process and to answer any questions.

The area covered by the demographic surveillance site is split into 21 reporting groups, which are in turn divided into 278 clusters. Each cluster has a key informant who lives in one of the villages covered and has been trained by the study staff to use formatted registers for recording and subsequently notifying staff of vital events and individuals who have migrated.^27^ For our contraception study, the study staff trained the 278 key informants to distribute family planning cards between January and April 2012. Thirty separate training sessions were held, during which the key informants were trained to use listings of the approximately 25 to 40 women of reproductive age living in each cluster. Then, each woman on each list was visited by a key informant who explained the study and asked whether the family planning card, which was preprinted with information identifying the woman, could be stapled to the inside front page of the woman’s health passport. The list was updated to reflect whether or not the woman accepted the family planning card. The list was returned, along with any remaining family planning cards, to the study staff at a meeting held roughly 10 days after the initial training. Key informants received a small payment in recognition of their efforts. The educational level, skills and age of key informants varied. For those key informants who struggled with the task, a study staff member either visited them to assist or matched the key informant with another who had demonstrated competency so they could work together.

The 132 health-care providers working in the area were trained in six separate sessions to record the following information on the family planning cards whenever they provided contraceptive services: (i) the date of the visit; (ii) the method of contraception received or the advice given; (iii) the provider’s individual 3-digit staff code; and (iv) where the service was delivered. Three refresher training sessions were conducted for all health-care providers and they received five motivational text messages in July, October and November 2012 and January and March 2013, respectively, to encourage them to continue recording information on the cards. The Karonga district family planning coordinator designated this task as part of health-care providers’ record-keeping responsibilities. All providers were given prepaid mobile phone units so they could call the study team if they had questions.

An interim audit of 379 family planning cards, which did not involve collecting the cards, was carried out six months after they were issued to determine whether the study methods were working and whether health-care providers were recording data on the cards. Data from this exercise are not presented here but study leaders were satisfied that the field work was progressing successfully.

After one year of data collection, the family planning cards were collected by the study staff at prearranged locations between February and May 2013. Efforts were made to locate women who had moved during the study year using up-to-date migration information from the prevention study database to minimize the number lost to follow-up. The study staff added any missing contraceptive data by checking the woman’s health passport to determine if any event recorded elsewhere in the passport was omitted from the family planning card and by asking her about family planning visits made during the previous year. The information source for each contraceptive event (i.e. family planning card, health passport or verbal report) was also recorded. Consequently, most data were collected prospectively – they were based on written reports by health-care providers. However, some gaps in data were filled in retrospectively from the women’s verbal reports. If a woman had undergone tubal ligation or had received a contraceptive implant or an intrauterine device before receiving the family planning card, this was recorded as an explanation of why the card was blank.

At the time of data collection, each woman provided individual, informed, written consent for the data to be used in the analysis. Consent was not requested at the beginning of the study because the key informants were not members of the Karonga prevention study staff and could not administer the informed consent procedure. Women were compensated with a small payment for the time required to attend the data collection session. A small number of family planning cards were gathered after data collection had officially finished either as part of a three-month, opportunistic, mopping-up operation or whenever demographic surveillance site field staff encountered a woman who had not returned her card.

### Statistical analysis

Data were managed and analysed using Stata version 12 (StataCorp. LP, College Station, United States of America). Differences in descriptive data between study participants and non-participants were evaluated using the *χ^2^* test to determine if there was any selection bias. 

## Results

**Fig. 1:**
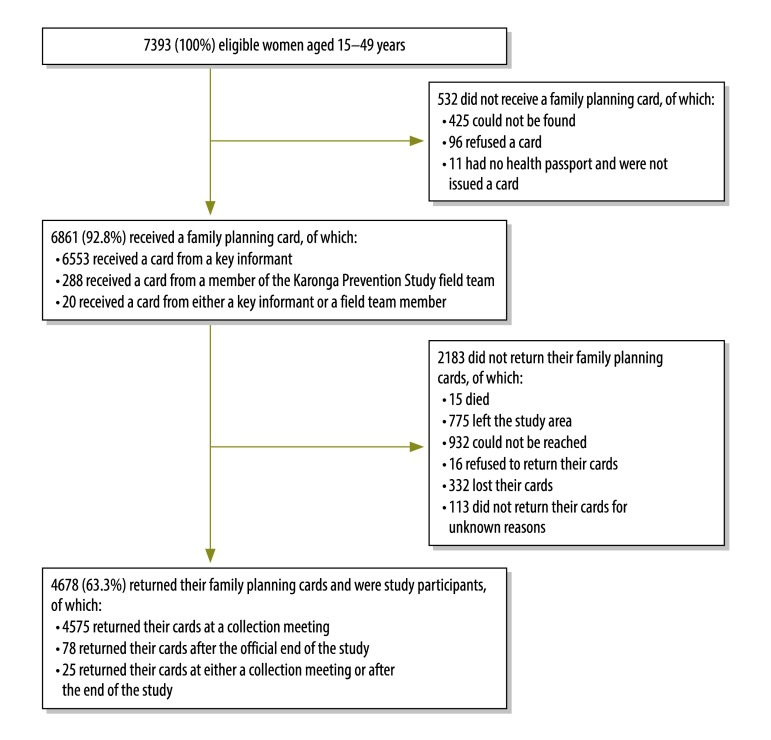
Participants in the Karonga family planning study, Malawi, 2012–2013

Note: Inconsistencies arise in some values due to rounding.

Details of study recruitment are presented in Fig. 1. In total, 6861 of the 7393 (92.8%) eligible women were issued with a family planning card. The proportion of women who received a card was highest in those older than 40 years (95.7%; 1071/1119) and lowest in those younger than 20 years (89.7%; 1480/1650). Most cards (95.7%; 6553/6841) were issued by key informants. The main reason for not issuing a card was that the woman could not be found (5.7%; 425 of eligible women) and actual refusals were infrequent (1.3%; 96). By the end of the one-year observation period, 2183 (31.8%) family planning cards had not been collected. The most common reasons were that the woman could not be found (13.6%; 932) or that the woman left the study area during the study period (11.3%; 775). In addition, 4.8% (332) lost their cards during follow-up and could not be included in the analysis. Although the majority of women returned their cards at prearranged collection meetings (4575/4653; 98.3%), a few gave their cards to the study staff at a later date. Overall, family planning cards were collected from 4678 women – 63.3% of all women eligible to participate in the study.

The socioeconomic characteristics and HIV status of all women eligible to participate in the study, of women who received a family planning card and of women who returned their family planning cards are described in Table 1. The characteristics of women who returned their family planning cards (i.e. study participants) were compared with those of women who either did not return their cards or did not receive a card to determine if there was any bias in recruitment. It was found that study participants were older, had a lower educational level and were more likely to be married, to want no more children and to have had five or more children than non-participants (*P <* 0.001 for all). There was no difference in HIV status. In addition, the characteristics of women who returned their cards at prearranged collection places were compared with those of women who returned their cards after formal data collection had been completed. There was no significant difference in marital status, educational level, HIV status, parity or childbearing intentions between the two groups (*P* > 0.001; data available from corresponding author, which indicates that the method of retrieving the card did not introduce a bias into the sample).

**Table 1:** Women in the Karonga family planning study, Malawi, 2012–2013

Characteristic	Women eligible to participate (*n* = 7393)	Women who received a family planning card (*n* = 6861)	Participants^a^ (*n* = 4678)	Non-participants^b^ (*n* = 2715)	*P*^c^
**Age in years, mean**	29.0	29.2	30.4	26.6	< 0.001
**Marital status, No. (%)^d^**					< 0.001
Married	4923 (66.9)	4668 (68.3)	3419 (73.2)	1504 (55.8)	
Separated, widowed or divorced	993 (13.5)	909 (13.3)	616 (13.2)	377 (14.0)	
Never married	1446 (19.6)	1258 (18.4)	633 (13.6)	813 (30.2)	
Total	7362 (100)	6835 (100)	4668 (100)	2694 (100)	
**Educational level, No. (%)^d^**					< 0.001
Did not complete primary school	548 (7.4)	524 (7.7)	355 (7.6)	193 (7.1)	
Completed primary school	4203 (57.0)	3980 (58.1)	2870 (61.4)	1333 (49.3)	
Secondary school or higher	2628 (35.6)	2345 (34.2)	1449 (31.0)	1179 (43.6)	
Total	7379 (100)	6849 (100)	4674 (100)	2705 (100)	
**HIV status, No. (%)^d^**					0.916
Positive	572 (8.9)	532 (9.0)	369 (8.9)	203 (9.0)	
Negative	5824 (91.1)	5349 (91.0)	3770 (91.1)	2054 (91.0)	
Total	6396 (100)	5881 (100)	4139 (100)	2257 (100)	
**Parity, No. (%)^d^**					< 0.001
None	183 (3.4)	169 (3.3)	102 (2.7)	81 (5.0)	
1–4	3328 (61.4)	3141 (61.0)	2235 (58.8)	1093 (67.6)	
≥ 5	1905 (35.2)	1837 (35.7)	1463 (38.5)	442 (27.4)	
Total	5416 (100)	5147 (100)	3800 (100)	1616 (100)	
**Childbearing intention, No. (%)^d^**					< 0.001
No more children	2110 (42.1)	2023 (42.8)	1559 (45.7)	551 (34.4)	
Child wanted after two years or more	1896 (37.8)	1772 (37.5)	1215 (35.6)	681 (42.5)	
Child wanted within two years	638 (12.7)	596 (12.6)	435 (12.7)	203 (12.7)	
Unsure	369 (7.4)	337 (7.1)	203 (5.9)	166 (10.4)	
Total	5013 (100)	4728 (100)	3412 (100)	1601 (100)	

HIV: human immunodeficiency virus.

^a^ Participants were women who both received and returned a family planning card.

^b^ Non-participants were women who either did not return their family planning cards or did not receive a card.

^c^ Participants versus non-participants.

^d^ Percentages are of the total number of women with data available on the characteristic.

Note: Inconsistencies arise in some values due to rounding.

To evaluate the study methods, we examined the information source for each contact between a health-care provider and a woman that concerned tubal ligation, a contraceptive implant, an intrauterine device, injectable contraceptives or oral contraceptive pills. In total, there were 3122 contacts and the source of most data was the information recorded by health-care providers on either the family planning card (78.3%; 2444) or the health passport (9.0%; 281; Table 2). Nevertheless, 12.7% (397) of provider–woman contacts were not recorded on a paper health record but were reported retrospectively by women during supplementary verbal interviews. Health surveillance assistants and volunteer community-based distribution agents were the most diligent at recording information on the family planning cards: 80.2% (1653) of contacts with a health surveillance assistant were recorded, as were 81.0% (277) of contacts with a community-based distribution agent. Clinical officers were least likely to record contacts: only 58.3% (91) of contacts were recorded on family planning cards and only 9.0% (14) on health passports. Receipt of male or female condoms was predominantly reported verbally by the woman rather than recorded by the health-care provider on the family planning card or health passport. In Malawi, these items can be obtained from sources other than health-care providers.

**Table 2:** Information source for each contact between a health-care provider and a woman, Karonga family planning study, Malawi, 2012–2013

Health-care provider	Information source for each contact, No. (%)			
	Family planning card	Health passport	Verbal report by woman	Total
Clinical officer	91 (58.3)	14 (9.0)	51 (32.7)	156 (100)
Medical assistant	31 (72.1)	7 (16.3)	5 (11.6)	43 (100)
Nurse	386 (75.0)	78 (15.1)	51 (9.9)	515 (100)
Health surveillance assistant	1653 (80.2)	177 (8.6)	230 (11.2)	2060 (100)
Community-based distribution agent	277 (81.0)	5 (1.5)	60 (17.5)	342 (100)
Youth community-based distribution agent	6 (100)	0 (0)	0 (0)	6 (100)
**Total**	**2444 (78.3)**	**281 (9.0)**	**397 (12.7)**	**3122 (100)**

Note: The contraceptive services provided included tubal ligation, contraceptive implants, intrauterine devices, injectable contraceptives and oral contraceptive pills.

## Discussion

Here we used an approach for collecting data on contraception based on patient-held records. Our experience may be useful to others who wish to conduct similar prospective research, particularly where there is a demographic surveillance site that can provide detailed and reliable data on the personal and family characteristics of potential users of family planning services. Our method employed several strategies for distributing and collecting family planning cards that enabled prospective data on contraceptive use to be gathered relatively quickly and economically from a very large number of women. With conventionally collected data on contraception, it is usually not possible either to track women over time or to link the services received from different facilities and providers. Findings using our approach will be presented in future research articles and will focus, in particular, on the continuity of contraceptive use by women and on providing more accurate estimates of contraceptive use that are not subject to the risk of overreporting.

During our study, the close working relationship between the study staff and the district health office ensured a good rapport between the study staff and the health-care providers responsible for recording data. We found that repeated refresher training and reminder text messages served well to motivate health-care providers and keep them engaged with the study and may have reduced underreporting. The observation that higher-level health-care providers were less diligent at recording data than lower-level providers is a phenomenon that has been reported elsewhere.^28^^,^^29^

There was some evidence of selection bias, which was introduced during recruitment and due to factors associated with not returning the card. This has implications for the way contraception data should be interpreted: study participants were slightly older and more likely to be married than non-participants and it is known that older, married women are more likely to be contraceptive users.

Most data were collected prospectively on family planning cards or health passports that were completed by health-care providers even though they were busy and had not been trained to carry out research. The fact that some data were not recorded on paper records at the time the contraceptive service was provided has general implications for the credibility of routine health data. Moreover, missing data make it difficult for health-care providers to offer a consistent service as they are unable to see details of previous contraceptive services provided to their patients. The use of existing patient-held records as the sole source of data on the continuity of contraceptive use, therefore, has limitations in the absence of strengthened systems for routine data collection. Fortunately, for the purposes of our study, gaps in the data recorded by health-care providers were filled in by the study staff who interviewed women about their contraceptive encounters when they collected the family planning cards. The result was a more complete data set than can be achieved using conventional data collection methods.

Several lessons can be learnt from our experience with this approach to data collection. In Malawi, some new health passports include a section akin to the family planning card where the use of oral contraceptive pills or injectable contraceptives can be recorded. Service providers could be trained to fill in this section correctly, perhaps with the aid of techniques such as reminder text messages. Periodic sampling of these records would provide longitudinal data on contraceptive use that could be used to analyse switching and discontinuation. The marginal additional cost of collecting contraceptive data on top of existing demographic surveillance site costs was 2.33 United States dollars per card per year. Better use of the family planning section of health passports and periodic sampling could be achieved if their costs were incorporated into the cost of providing health passports and routine data collection.
